# The association between growth patterns and blood pressure in children and adolescents: A cross‐sectional study of seven provinces in China

**DOI:** 10.1111/jch.14393

**Published:** 2021-11-30

**Authors:** Manman Chen, Ying Ma, Tao Ma, Yanhui Li, Di Gao, Li Chen, Jieyu Liu, Yi Zhang, Jun Jiang, Xinxin Wang, Yanhui Dong, Jun Ma

**Affiliations:** ^1^ School of Public Health National Health Commission Key Laboratory of Reproductive Health Institute of Child and Adolescent Health Peking University Beijing China; ^2^ Department of Plant Science and Landscape Architecture University of Maryland College Park Maryland USA; ^3^ School of Public Health and Management Key Laboratory of Environmental Factors and Chronic Disease Control Ningxia Medical University Ningxia China

**Keywords:** birth length, height, growth patterns, blood pressure, growth patterns, children and adolescents

## Abstract

Aimed to investigate the associations between different growth patterns with high blood pressure, and further examine the mediation effect of BMI between growth patterns and high blood pressure among children and adolescents. A total of 31581 children and adolescents aged 7–18 years were selected based on the stratified cluster sampling method. Logistics regression models were used to calculate the odds rations (ORs) and 95% confidence interval (95%CI) of the association between different growth patterns and high blood pressure. Mediation effect analyses were applied to estimate the effect of BMI on the increase of blood pressure levels in different growth patterns. In different sex and ages, compared to reference group of normal growth, blood pressure levels and prevalence of high blood pressure of the catch‐up growth were higher, but that of the catch‐down growth were lower. The prevalence of high blood pressure was 11.69%, 16.06%, and 9.68% in normal growth, catch‐up growth, and catch‐down growth, respectively. In total, compared with the normal growth pattern, the ORs (95%CI) of high blood pressure, high systolic blood pressure and high diastolic blood pressure in the catch‐up growth were 1.171(1.073,1.280), 1.110(1.001,1.230) and 1.141(1.025,1.270) (*p *< .05), respectively. Additionally, the mediation effect of current BMI existed in the association between blood pressure levels and different growth patterns, particularly in boys. Our findings suggested that different growth patterns after birth could modify blood pressure, and the potential risks of high blood pressure could be increased by catch‐up growth at childhood and adolescence.

## 1 INTRODUCTION

High blood pressure (HBP) during childhood and adolescence has become a major public health problem in the world.^1^ The global prevalence of HBP in children and adolescents was on a long‐term upward trend with obesity pandemic, particularly in Asian countries.^2^, ^3^ One study conducted in US reported the prevalence of HBP was 16.3% in 2017 among adolescents aged 10–17 years.^4^ Similar in China, the prevalence of HBP in children and adolescents fluctuated between 6.9% and 9.2% from 1995 to 2014 based on a successive national survey.^5^, ^6^ The BP levels of children and adolescents should always be a good predictor of adult HBP with the phenomenon of “trajectory”, that was often overlooked.^7‐9^ Therefore, HBP that once was considered a rare disease in children is now actually a major public health problem worldwide,^10^ due to the tracking of risks with organ damages including coronary artery calcifications and hypertrophy from childhood to adulthood.^11^, ^12^

Simultaneously, there were many factors that could affect HBP in children and adolescents, such as family history, physical exercise, diet and body fat.^13^ Among the multiple factors, body types, like weight and height, has always been confirmed to be one of the leading drivers to HBP.^14^, ^15^ Notably, unlike weight with much attention, height was also a strong and independent indicator in determining the increases in BP, since the definition of HBP among children and adolescents was comprehensively determined by age, sex, and height in the current international criteria.^16^, ^17^, ^18^ However, the effect of growth patterns measured by the indicator of height on BP during childhood and adolescence was still unknown.

Some indicators of infants followed a regular growth patterns after birth, such as body length, height, weight, body mass index (BMI), and head circumference.^19^ Under various nutrition environments, they were outgrowing, or falling behind, or still following the established trajectory.^20^ A study from US divided growth patterns into catch‐down growth, normal growth and catch‐up growth,^21^ which caused widespread. In each stage of growth in children, they might suffer from diseases, malnutrition, psychological stress, and other adverse events, which would cause the phenomenon of catch‐down growth.^22^, ^23^ Catch‐down growth, is often observed in babies born at intrauterine growth restriction and small for gestational age.^24^, ^25^, ^26^ Once these factors above were removed, the height growth of children would catch up the original normal trajectory. This phenomenon of accelerated growth was called catch‐up growth pattern.^20^, ^27^ Thus, catch‐up growth was defined as “a height velocity above the statistical limits of normality for age or maturity during a defined period of time, following a transient period of growth inhibition; the effect of catch‐up growth is to take the child towards his/her pre‐retardation growth curve”.^28^, ^29^

Many studies reported the adverse events of catch‐down growth of children with linear growth failure, including multiple pathological disorders associated with increased morbidity and mortality, loss of physical growth potential, nutritional diseases, and lung diseases in adulthood.^30^, ^31^ Previous studies also showed that children with catch‐up growth could have a higher risk of cardiovascular and metabolic diseases later in life than those with normal growth patterns.^32^ In addition, the strong association between BP and BMI had been well documented in children and adolescents,^33^ and previous studies had explored potential mechanisms linking growth patterns and cardiovascular diseases.^34^ However, few studies explored the associations between different growth patterns and HBP risks among children and adolescents, and given the importance of BMI, the mediation effect of BMI on the increase of BP levels among different growth patterns is still lacking.

Thus, using the data from a national representative survey in Chinese seven provinces, the present study aimed to determine the association between different growth patterns and HBP risks among children and adolescents, and to explore the potential mediation effect of BMI between growth patterns and BP levels.

## 2 METHODS

### 2.1 Design and participants

**FIGURE 1:**
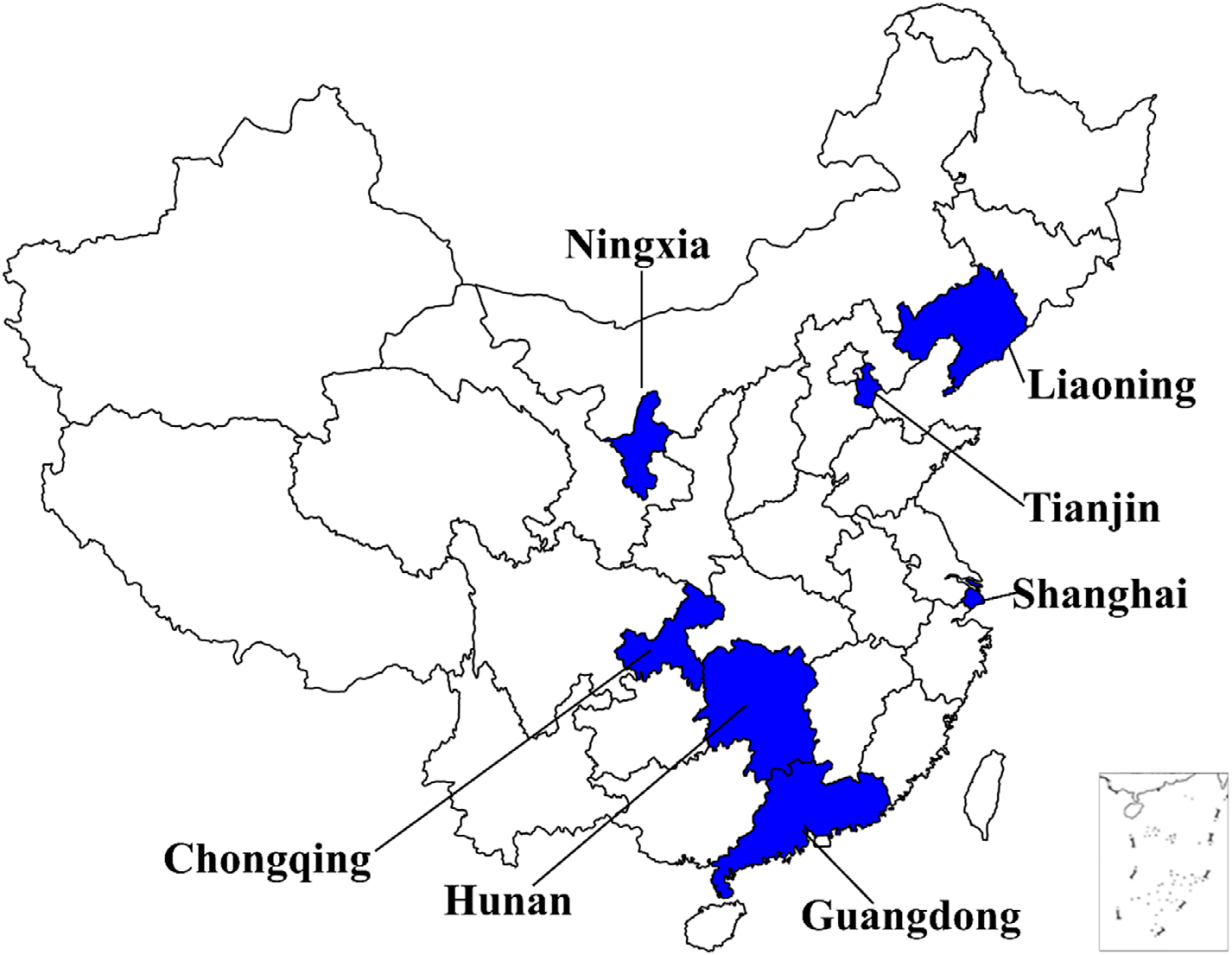
Locations of the seven provinces of survey in China

The data in this study came from 93 primary and secondary schools in seven provinces, including Guangdong, Hunan, Liaoning, Shanghai, Chongqing, Tianjin, and Ningxia (**Figure** **1**). The study adopted the multistage cluster random sampling, which has been published in detail in previous studies.^35^ Briefly, one city was selected in each of seven provinces according to their economic level. In each selected city, about 16 primary and secondary schools were selected. Among the selected schools, two classes per grade were randomly selected with all students aged 6–18 years considered eligible. All eligible participants in our study underwent a complete medical examination before data collection and those were excluded if they had one or more of the following conditions: (1) serious organ disease or consumed stimulating drugs; (2) abnormal physical development; (3) physical impairment or deformity; (4) acute disease symptoms during the past month; or (5) very low birthweight infants (< 1500 grams). A total of 3258 observations (8.39%) were removed because of missing data or extreme height, weight, and BP values (> 5 SDs of sex‐ and age‐specific mean). Finally, the study involved 31,581 children and adolescents aged 6–18 years (15853 boys and 15728 girls) with complete individual information, such as age, sex, birth date, birth length, birth weight, height, weight, and BP. The research content included general body measurement and questionnaire survey. Informed consent was signed by all patients and their parents. This study was approved by the Medical Research Ethics Committee of Peking University Health Science Center (IRB00001052‐13034).

### 2.2 Measurements

The measurement of each anthropometric index, including height (cm), weight (kg), BP (mm Hg), in our study followed a standardized procedure by professionals who had passed the training course, details of which have been published previously.^36^ The standardized measurement procedure referred to the anthropometry methods in the 2006 WHO Child Growth Standards for height and weight (http://www.who.int/childgrowth/ standards/en/), and new clinical practice guideline on high BP in childhood by American Academy of Pediatrics (AAP) in 2017.^37^ In short, all anthropometric indexes were measured twice and taken the average values.

Height was measured two times using a uniform and calibrated mechanical stadiometer (model TZG, China), with an accuracy of 0.1 centimeter. At the same time, the participants stood straight without shoes, their heels were together with toes apart at a 60^o^ angle, and their backs were against a calibrated backboard. The trained staff slid the horizontal slab down to the top of the children's head with one hand, and keep the eyes and the level of the horizontal slab at the same height when they were reading. Weight was also measured twice with a uniform and calibrated electronic scale (model RGT‐140, China) to the nearest 0.1 kilogram while patients were wearing short clothes and standing naturally in the center of the weight measuring plate to keep the body stable. Weights were recorded with waiting for the value on the scale display to stabilize. According to the instructions for use, checked its working status, accuracy and sensitivity before used. Height and weight were calculated the average of two repeated measurements. In rechecking 10% of the students, the error was less than 5%. BMI was calculated as (weight(kg)/height(m)^2^), and the age was calculated as (examine date‐birthday)/365.25.

The BP levels were based on auscultatory measurements by the National High Blood Pressure Education Program Working Group in Children and Adolescents,^17^ adopting mercury sphygmomanometers (model XJ11D, China) and stethoscopes (model TZ‐1, China) with an appropriate cuff size. Before the measurement of our study, participants should have avoided stimulant drugs to control the BP levels, and have been sitting quietly still for about 5–10 min. Systolic blood pressure (SBP) was determined by onset of the first Korotkoff sound and diastolic blood pressure (DBP) was determined by the fifth Korotkoff sound. BP was measured twice with a 5 min gap between the two measurements from the right arm. If the difference between two measurements was 10 millimeters of mercury or higher, the third measurement would be conducted until the difference was less than 10 mm Hg, then the two closest values were adopted, and the average of SBP and DBP values were calculated, respectively.

Birth length data was collected by a standard questionnaire survey. Parents needed to record their children's birth length according to their birth certificate or health clinic record. If they did not have it, they were asked to recall their children's data. All of the birth length was measured by trained nurses and recorded to the nearest 0.1 cm when they were born. About 75% of parents recorded the data of their children's birth length based on the birth certificate or health clinic card. During the entire research process, to ensure the reliability of birth length, parents were asked to repeat the same process of questionnaire survey twice, if the difference was more than 2 cm, we judged the sample as missing.

### 2.3 Definitions of growth patterns

Participants’ birth length and height were Z‐Scored according to the 2000 Centers for Disease Control and Prevention growth charts for the United States (US CDC standards (2000)),^21^ and it was recommended to assess size and growth in infants, children, and adolescents in clinical practice.^38^ According to the definition by Robert J. Kuczmarski, and his colleagues, a gain in Z‐Score for birth length and current height greater than 0.67 was taken to indicate clinically significant catch‐up growth, similarly, a decrease in Z‐Score less than ‐0.67 was considered as catch‐down growth, and the others between the two limits were judged as normal growth.

### 2.4 Definitions of HBP

BP levels were divided according to the Clinical Practice Guideline for Screening and Management of High Blood Pressure in Children (2017 Guideline).^16^ Normal BP was defined as SBP and DBP that were < 95th percentile for sex, age, and height. HSBP (high systolic blood pressure) and HDBP (high diastolic blood pressure) were defined as SBP or DBP greater than or equal to the 95^th^ age‐, sex‐, and height‐ specific percentile for children younger than 13 years, and greater than or equal to 130/80 mm Hg for children and adolescents aged 13 years or older.^16^ Participants with at least HSBP or HDBP were defined as having HBP. In addition, according to the National High Blood Pressure Education Program (NHBPEP) Working Group on HBP in Children and Adolescents (2004),^39^ the Z‐Score of SBP and DBP by sex, age, and height was obtained.

### 2.5 Questionnaire data collection

Before conducting the survey, the spreads were carried out, and all the questionnaire questions were strictly checked. Participants’ sociodemographic variables and basic information were collected by questionnaire survey. Child‐reported questionnaires were filled in by students in the classroom, with interpretation performed by professional investigators, with the exception of children at third grade or under primary school, who completed the questionnaires at home with their first guardian. The age was calculated as (examine date‐birth date)/365.25, and we divided the age into three groups (6–12years, 13–15years and 16–18years). Generally, these three age groups represented the primary school stage (grade 1–6), middle school stage (grade 1–3), middle high school stage (grade 1–3) in China. Child‐reported questionnaires collected included birth date, sex, area (urban/rural, for the past year), daily moderate physical activity (calculated as average daily time = (days×(time in each of those days))/7), and etc. The parent‐reported questionnaire contained the child's and parent's information, including (height, weight, first steatorrhea(yes/no), menarche(yes/no), breastfeeding (yes/no, lasting for over a month), family history of hypertension (yes/no, either parent had can be judged as yes), father and mother education (1 = high school and below, 2 = junior college and above).

### 2.6 Statistical analysis

Continuous variables were expressed by mean values and standard deviations, and categorical variables were expressed by numbers and percentages. Chi‐square test was used to analyze characteristics of participants in different growth patterns and the association between different growth patterns and BP levels. Sex‐specific multivariable logistic regression models were used to analyze the risks between different growth patterns and HBP, HSBP and HDBP with to odds ratio (OR) and 95% confidence interval (95%CI). In order to adjust the confounding factors (age group, area, birthweight, first steatorrhea, menarche, parental education, parental BMI, family history of hypertension, breast feeding, daily moderate physical activity), two models were conducted in the calculation. Model1 was adjusted for basic and sociodemographic information, model2 was adjusted the greater impact on BP. In model 1, some confounders were adjusted including age, urban/rural living condition, birthweight, first steatorrhea, menarche, father and mother education (high school and below, junior college and above), father BMI, mother BMI, family history of hypertension (yes/no), breast feeding (yes/no), and daily moderate physical activity (hours). In model 2, current BMI was adjusted based on model 1.

BMI had been proven to have a greater impact on BP, and needed to be further verified. To understand the mediation role of BMI on the increase of BP levels in different growth patterns, we used linear regressions to analyze associations (total association, c coefficient) between variable X (e.g., Growth patterns) and variable Y (e.g., SBP, DBP). Thereafter, we used same methods to analyze associations (a coefficient) between X (e.g., Growth patterns) and potential mediators Z (e.g., BMI). Then, associations between potential mediators Z (e.g., BMI) and dependent variable Y (e.g., SBP, DBP) were examined (b coefficient). Finally, if c, a and b coefficients were all statistically significant, the mediation effect of potential mediator Z on the association between X and Y was proved. Additionally, the proportion of mediation was calculated. Statistical analysis was performed using Stata version 14.0, we considered the associations to be significant when the two‐sided *p* value was less than .05.

## 3 RESULTS

### 3.1 General demographic characteristics

Characteristics of participants in different growth patterns were shown in **Table** **1**. Among the 31581 children and adolescents, there were 15853(50.20%) boys and 15728(49.80%) girls. The proportion of normal growth, catch‐up growth and catch‐down growth among children and adolescents were 40.43%, 40.76%, and 18.82%, respectively. Under different growth patterns, there was a difference in the ratio of boys to girls. Participants in catch‐up growth pattern tended to have a lower birth length, only 49.42 cm, while the figure of those in catch‐down growth pattern reached at 52.59 cm. Similar trend was observed for the birth weight. The current BMI value of normal growth, catch‐up growth and catch‐down growth pattern was 18.16 kg/m^2^, 18.88 kg/m^2^ and 18.32 kg/m^2^ (*p *< .001), respectively. Differences in age group, areas, breast feeding, parental educational and parental BMI between growth patterns groups were detected.

**TABLE 1:** Demographic characteristics of participants in different growth patterns

			Growth patterns groups				
Variables		Total	Normal	Catch‐up	Catch‐down	*χ2*/*F*	*P*
Sample, *n* (%)		31581	12767 (40.43%)	12871 (40.76%)	5943 (18.82%)	−−	–
Birth time, (Mean ± SD)	birth length, cm	50.52 ± 2.26	50.66 ± 1.76	49.42 ± 1.96	52.59 ± 2.29	5431.748	<.001
	birth weight, kg	3.31 ± 0.48	3.32 ± 0.48	3.28 ± 0.49	3.36 ± 0.48	52.976	<.001
	birth length Z	−0.17 ± 0.80	−0.11 ± 0.61	−0.57 ± 0.68	0.59 ± 0.80	5992.595	<.001
	birth weight Z	−0.10 ± 0.90	−0.09 ± 0.88	−0.15 ± 0.91	−0.01 ± 0.88	24.865	<.001
Present time, (Mean ± SD)	height, cm	144.35 ± 16.73	143.08 ± 16.88	146.41 ± 16.6	142.62 ± 16.24	167.509	<.001
	weight, kg	40 ± 15.46	38.67 ± 15.16	41.9 ± 16.03	38.71 ± 14.40	167.133	<.001
	BMI, kg/m^2^	18.49 ± 3.84	18.16 ± 3.73	18.88 ± 3.99	18.32 ± 3.64	119.571	<.001
	height Z	0.21 ± 0.99	−0.06 ± 0.65	0.95 ± 0.78	−0.81 ± 0.79	13211.1	<.001
	weight Z	0.30 ± 1.29	0.01 ± 1.03	0.92 ± 1.40	−0.44 ± 0.86	3388.401	<.001
	BMI Z	0.21 ± 1.31	0.04 ± 1.20	0.52 ± 1.46	−0.13 ± 1.00	703.703	<.001
Sex, *n* (%)	boys	15853	6135 (48.05)	7359 (57.18)	2359 (39.69)	536.410	<.001
	girls	15728	6632 (51.95)	5512 (42.82)	3584 (60.31)		
Areas, *n* (%)	urban	18543	7434 (58.23)	7943 (61.71)	3166 (53.27)	121.566	<.001
	rural	13038	5333 (41.77)	4928 (38.29)	2777 (46.73)		
Age group, *n* (%)	6‐12years	22336	8856 (69.37)	10079 (78.31)	3401 (57.23)	975.334	<.001
	13‐15years	6396	2640 (20.68)	2114 (16.42)	1642 (27.63)		
	16‐18years	2849	1271 (9.96)	678 (5.27)	900 (15.14)		
Breast feeding, *n* (%)	yes	26482	10787 (85.11)	10583 (82.82)	5112 (86.69)	52.710	<.001
	no	4867	1887 (14.89)	2195 (17.18)	785 (13.31)		
Family history, *n* (%)	yes	2128	826 (6.70)	928 (7.46)	374 (6.50)	8.883	.064
	no	28392	11497 (93.30)	11517 (92.54)	5378 (93.50)		
Father BMI, *n* (%)	normal	16012	6710 (52.56)	5951 (46.24)	3351 (56.39)	223.213	<.001
	overweight	11930	4702 (36.83)	5175 (40.21)	2053 (34.54)		
	obesity	3639	1355 (10.61)	1745 (13.56)	539 (9.07)		
Mother BMI, *n* (%)	normal	24318	9967 (78.07)	9775 (75.95)	4576 (77.00)	16.715	.002
	overweight	5959	2303 (18.04)	2530 (19.66)	1126 (18.95)		
	obesity	1304	497 (3.89)	566 (4.40)	241 (4.06)		
Father education, *n* (%)	high school and below	21344	8677 (67.96)	8182 (63.57)	4485 (75.47)	264.11	<.001
	junior college and above	10237	4090 (32.04)	4689 (36.43)	1458 (24.53)		
Mother education, *n* (%)	high school and below	22009	8915 (69.83)	8442 (65.59)	4652 (78.82)	310.040	<.001
	junior college and above	9572	3852 (30.17)	4429 (34.41)	1291 (21.72)		

*Note*: Continuous variables were expressed by mean values and standard deviations (Mean ± SD), and categorical variables were expressed by numbers and percentages (*n* (%)).

### 3.2 Associations of BP Z‐Scores with birth length, height, and the difference Z‐Scores

**FIGURE 2:**
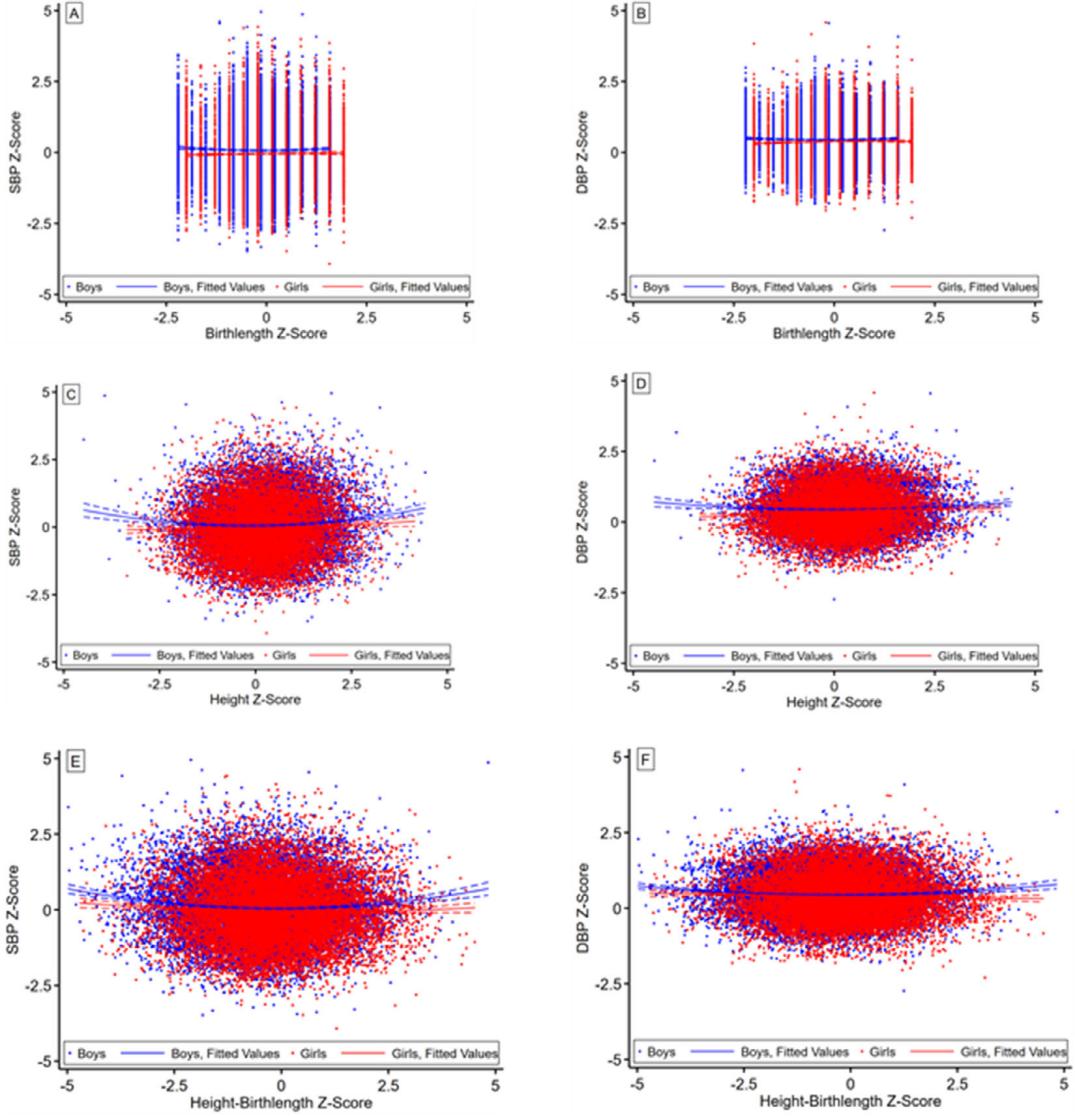
Associations of BP Z‐Scores with birth length, height and the difference Z‐Scores in children and adolescents by sex

A scatter diagram and its fitted lines showed the visual association between the SBP and DBP Z‐Scores with birth length, height, and the difference Z‐Scores among boys and girls (**Figure** **2**). We found that the DBP Z‐Scores was positively associated with birth length Z‐Scores (*r *= 0.022, *p *= .006). Apart from this, a significant U‐shaped relationship between both SBP and DBP Z‐Scores with the current height Z‐Scores was observed, and the same results also existed between both SBP and DBP Z‐Scores and the difference of current height and previous birth length Z‐Scores.

### 3.3 The levels of BP by sex and age group

**FIGURE 3:**
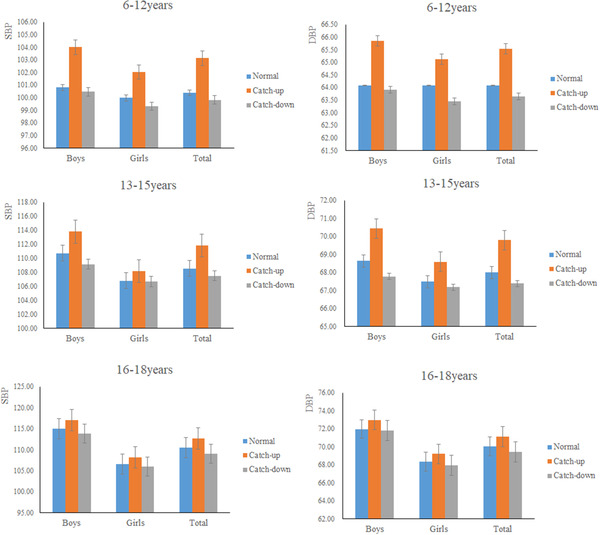
The levels of BP along with different growth patterns, by sex and age

BP levels of catch‐up growth were higher than that in other two growth patterns groups (**Figure** **3**). Furthermore, boys were more likely to have higher BP levels than girls. Both SBP and DBP levels were increased with aging. For example, in the catch‐up growth group, participants aged 16–18 years old had the highest BP levels, among which boys’ BP (117.05 mm Hg) was significantly higher than that of girls (108.19 mm Hg). Consistent trend was detected for DBP values. Notably, the levels of BP in catch‐down growth group were lower than that of the normal growth group.

The prevalence of HBP, HSBP and HDBP among different growth pattern groups is displayed in **Table** **2**. The high prevalence of HBP was found, with 11.69%, 16.06%, and 9.68% in normal growth, catch‐up growth, and catch‐down growth, respectively. Obviously, the prevalence of HBP, HSBP and HDBP in catch‐up growth group was highest reaching at 16.06%, 11.08%, and 10.19%, respectively. In addition, there was statistically significance between different growth patterns and prevalence of HBP, HSBP, and HDBP (*p *< .001).

**TABLE 2:** Prevalence of HBP, HSBP and HDBP in different growth patterns among children and adolescents

Growth pattern	BP			SBP			DBP		
	Normal	HBP	*χ2*	Normal	HSBP	*χ2*	Normal	HDBP	*χ2*
Normal	11275(88.31)	1492(11.69)	182.775**	11730(91.88)	1037(8.12)	111.676**	11839(92.73)	928(7.27)	119.037**
Catch‐up	10804(83.94)	2067(16.06)		11445(88.92)	1426(11.08)		11559(89.81)	1312(10.19)	
Catch‐down	5368(90.32)	575(9.68)		5535(93.13)	408(6.87)		5584(93.96)	359(6.04)	
Total	27447(86.91)	4134(13.09)		28710(90.91)	2871(9.09)		28982(91.77)	2599(8.23)	

*Notes*: **p < .001.

*Abbreviations*: HBP, high blood pressure; HSBP, high systolic blood pressure; HDBP, high diastolic blood pressure.

### 3.4 Risk of different growth patterns and HBP

**Table** **3** showed the OR and 95% confidence intervals HBP, HSBP, and HDBP with different growth patterns in boys and girls. Compared to the reference group of the normal growth pattern, the OR (95% CI) of HBP, HSBP, and HDBP in catch‐up growth group significantly reached to 1.351(95% CI: 1.240,1.472), 1.302(95%CI: 1.178,1.438), and 1.290(95%CI: 1.161,1.443) (*p *< .001), respectively. In addition, similar findings were presented for both sex groups. In contrast, there was no significance between catch‐down growth pattern and risks of HBP, HSBP and HDBP in all participants. Furthermore, after adjustment for current BMI based on the model1, ORs of HBP, HSBP and HDBP reached to 1.171(95%CI: 1.073,1.280), 1.110(95%CI: 1.001,1.230) and 1.141(95%CI: 1.025,1.270) with significant difference to normal growth pattern group (*p *< .05). Additionally, we added the analysis of the Odds Ratios and 95% Confidence Intervals of different growth patterns and elevated blood pressure (Supplementary Table 1). Besides, we also analyzed the Odds Ratios and 95% Confidence Intervals of different growth patterns and High Blood Pressure among different ages (Supplementary Table 2).

**TABLE 3:** Odds Ratios and 95% Confidence Intervals of different growth patterns and High Blood Pressure

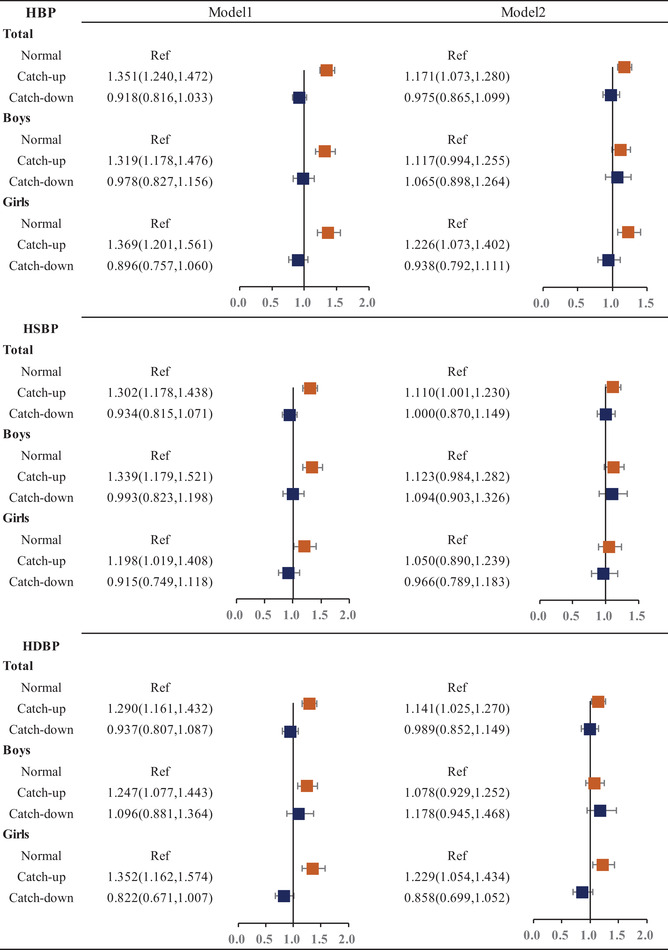

*Abbreviations*: HBP, high blood pressure; HSBP, high systolic blood pressure; HDBP, high diastolic blood pressure.

Model1: represents there were age, city, birthweight, first menstrual, gonacrat, father education, mother education, father BMI, mother BMI, family history of hypertension, breast feeding, daily moderate physical activity.; Model2: represents there were current BMI based on Model1.

### 3.5 Mediation of BMI on the increase of BP levels in different growth patterns

**FIGURE 4:**
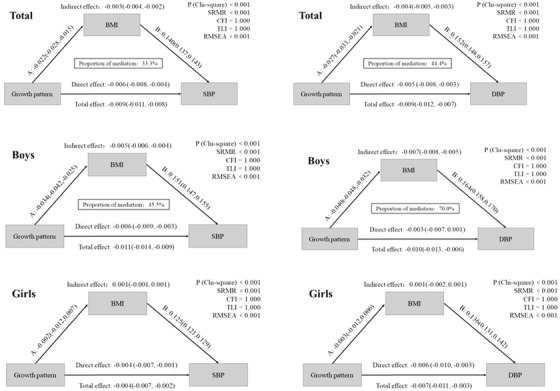
Analysis of the mediation role of BMI on the increase of BP levels in different growth patterns

**Figure** **4** showed the mediation effect of BMI on the increase of BP levels in different growth patterns for both sex. We found that the mediation effect existed, particularly in boys. Noticeably, among boys, there was a negative correlation between different growth patterns and the level of DBP, total effect was ‐0.010 (95%CI: ‐0.013, ‐0.006; *p *< .05). There was a negative correlation between different growth patterns and BMI (*β *= ‐0.040, 95%CI: ‐0.048, ‐0.032; *p *< .05), BMI and the level of DBP had a positive correlation (*β *= 0.164, 95%CI:0.158, 0.170; *p *< .05). Thus the mediation effect of BMI existed between different growth patterns and the level of DBP, with the value of ‐0.007 (95%CI: ‐0.008, ‐0.005; *p *< .05), and the percentage of 70.0%.

## 4 DISCUSSION

Our study mainly illustrated the associations between different growth patterns and BP levels among children and adolescents in China. The BP levels of catch‐up growth were higher than that of normal growth. After adjusted for confounders, compared with normal growth group, catch‐up growth pattern was positively associated with the risks of HBP, HSBP and HDBP, but the catch‐down growth pattern failed to reach significance. Furthermore, current BMI value could act as a mediating indicator in the relationship between the catch‐up growth patterns and HBP risks, especially among boys. This study with national large data of seven provinces provided an obvious evidence for guiding the scientific and reasonable growth and development of children and adolescents after birth with different growth patterns.

Compared to reference group of normal growth, BP levels and prevalence of HBP of the catch‐up growth were higher, but the that of the catch‐down growth were lower. Currently, catch‐up growth patterns are usually recommended, which can promote neurodevelopment and height growth. However, massive studies demonstrated that catch‐up growth patterns might lead to the high risk of obesity, insulin resistance, cardiovascular diseases and other adverse health outcomes.^40^, ^41^, ^42^ Besides, one study found that adults who were born with small for gestational age but experienced accelerated growth during growth and development had been considered to be at an increased risk of HBP,^43^ which was similar to our results. It was also found that insulin resistance and signal impairment in skeletal muscle inhibited thermogenesis during catch‐up growth, which could be the potential mechanisms of catch‐up growth with increased risk of HBP in later life.^44^, ^45^ There were some evidences that the state of insulin resistance in catch‐up growth would increase preferentially body fat content, which could induce metabolic diseases.^44^, ^46^ The possible mechanisms for this result were that the process of catch‐up growth could excessively consume the function of the early blood vessel wall, which affected the power of blood flow in the blood vessel and leads to increase in BP levels.^47^ The finding of this study manifested that cutch‐down growth had low BP levels, but had no substantial effect.

Until it was not clear that enabling catch‐up growth was of no cognitive benefit,^48^ specially, our study conducted that catch‐up growth could be a risk factor for HBP, HSBP and HDBP. The studies that investigated the association between catch‐up growth and SBP in adulthood all reported similar positive associations,^49^, ^50^ which supported the suggestion that small‐for‐gestational‐age infants, who tended to exhibit catch‐up growth, were at an increased risk of HBP.^51^ Instead, a more comprehensive guidance of HBP and catch‐up growth, which kept children and adolescents healthier after birth, should be more stressed. Noticeably, in the process of adjusting for current BMI, no statistically significant differences were observed between boys and girls. In addition, the mediation effect of BMI in different growth patterns on BP levels was only found in boys. The difference by sex might be attributed to the hormones. Considering that the serum estradiol and testosterone concentrations of prepubertal girls and boys were different, the estradiol level of prepubertal girls was much higher than that of prepubertal boys, and the serum testosterone concentration was the opposite.^52^, ^53^ Furthermore, androgens could cause cardiac hypertrophy, promote atherosclerosis, vascular remodeling and stimulate renal hypertension.^54^ Androgens seemed to play a role in the differentiation of brain regions involved in BP regulation, leading to vasodilation in boys with coronary artery disease, so long‐term exposure to androgens might trigger a variety of vasoconstriction mechanisms.^55^, ^56^ Additionally, it provided new theoretical basis and research ideas for the future identification and intervention of children and adolescents' overweight and obesity, and laid a foundation for further research on the relationship between different growth patterns and HBP.

Our research highlighted the significance of controlling BP on the health of the childhood after birth. The present study suggested that we might not blindly advocate the patterns of catch‐up growth, and we should pay close attention to the intensive changes in the growth process. What's more, when parents found that children were growing too fast, they should pay attention to control their diet, and guide them to have a healthy lifestyles and adequate physical exercise, to help to access catch‐up growth and potential risks of HBP timely. Therefore, the deleterious health problems during childhood and adolescence brought by catch‐up growth, such as adolescent obesity, hypertension, precocious puberty, and mental health, also deserve more attention and research.

Our study had obvious strengths that the national representative large sample size from seven provinces in China reflected the associations between different growth patterns and HBP risks in children and adolescents. However, several limitations should be noted. Firstly, birth weight and length were the core indicators of this study, therefore, we chose the information on the birth certificate as much as possible. Indeed, the results obtained in this way have a certain degree of bias, because there are measurement biases among nurses in different regions of the country. Another way collected by parents record according to their birth certificate or health clinic record, also existed recall bias. However, on this process we carried out strict quality control to ensure the reliability. Secondly, different growth patterns were defined by the Z‐Score of birth length and current height during a long interval, thus, the assessment of the growth pattern could be a bit rough, and it might neglect the detailed patterns in the middle of growth. In order to overcome the information of lacking of the Tana stage, we included these factors (first steatorrhea(yes/no), menarche(yes/no)) to replace the pubertal development status. Thirdly, our study was a cross‐sectional design, so we cannot conclude into a causal relationship, however, the infant birth length existed before the measurement of BP, an inferred cause association between growth patterns and BP levels should be reasonable. In addition, regarding the diagnosis of HBP, the standard we adopted was only a preliminary screening, but to some extent, our research was based on a large‐scale epidemiological survey, which could reduce the bias partially. Besides, we defined BP > 95% as HBP, which would reduce the strength of the correlation to a certain extent. A final limitation was that some potential confounders in our study were adjusted, but the risks of HBP could also be influenced by genetics and environment factors, which needed to be further explored and vitrificated in future study.

## 5 CONCLUSIONS

Catch‐up growth pattern was positively associated with higher risks of HBP, HSBP and HDBP in children and adolescents, but catch‐down growth patterns failed to reach significances. Current BMI value could act as a mediating indicator in the relationship between the catch‐up growth patterns and HBP risks, particularly in boys. Our study provided new theoretical basis and research ideas for the future identification and intervention of HBP among children and adolescents, and helped to assess potential risks of HBP and related metabolic diseases caused by catch‐up growth pattern after birth and at adulthood.

The study was supported by the Research Special Fund for Public Welfare Industry of Health of the Ministry of Health of China (1147 Project; Grant No. 20122010 to Jun Ma), China Postdoctoral Science Foundation (BX20200019 and 2020M680266 to Yanhui Dong) and National Natural Science Foundation of China (82103865 to Yanhui Dong).

## AUTHOR CONTRIBUTIONS

All authors contributed to conception and design of this study. Manman Chen and Ying Ma conceptualized and designed the study, did the statistical analyses, drafted and revised the manuscript. Manman Chen, Ying Ma, Tao Ma; Yanhui Li, Di Gao, Li Chen, Jieyu Liu, Yi Zhang, Jun Jiang and Xinxin Wang interpreted results, wrote and finalized the manuscript, Yanhui Dong and Jun Ma reviewed and revised the manuscript. All authors read and approved the final manuscript.

## CONFLICT OF INTEREST

The authors have no relevant financial or non‐financial interests to disclose.

## Supporting information

Supporting Information
