# Supplementary material for: The association between growth patterns and blood pressure in children and adolescents: A cross‐sectional study of seven provinces in China
Source: J Clin Hypertens (Greenwich). 2021 Nov 30;23(12):2053–64. doi: 10.1111/jch.14393 (PMC8696227; doi:10.1111/jch.14393)
Supplement: Supplementary file 1 — Supporting Information [file JCH-23-2053-s001.docx]

**Supplementary Table1: Odds Ratios and 95% Confidence Intervals of different growth patterns and Elevated Blood Pressure (Supplementary Table1).**

Notes: EBP, elevated blood pressure; ESBP, elevated systolic blood pressure; EDBP, elevated diastolic blood pressure.

Model1: represents there were age, city, birthweight, first menstrual, gonacrat, father education, mother education, father BMI, mother BMI, family history of hypertension, breast feeding, daily moderate physical activity.; Model2: represents there were current BMI based on Model1.

**Supplementary Table2: Odds Ratios and 95% Confidence Intervals of different growth patterns and High Blood Pressure among different ages (Supplementary Table2).**

Notes: HBP, high blood pressure;

Model1: represents there were age, city, birthweight, first menstrual, gonacrat, father education, mother education, father BMI, mother BMI, family history of hypertension, breast feeding, daily moderate physical activity.; Model2: represents there were current BMI based on Model1.
